# Comparison between manta trawl and in situ pump filtration methods, and guidance for visual identification of microplastics in surface waters

**DOI:** 10.1007/s11356-019-07274-5

**Published:** 2019-12-18

**Authors:** Therese M. Karlsson, Anna Kärrman, Anna Rotander, Martin Hassellöv

**Affiliations:** 1 Department of Marine Sciences, University of Gothenburg, Kristineberg 566, 45178 Fiskebäckskil, Sweden; 2 MTM Research Centre, School of Science and Technology, Örebro University, Orebro, Sweden

**Keywords:** Microplastics, Surface water sampling, Monitoring, Particle quantification, Method development, Plastic pollution, Microlitter

## Abstract

Owing to the development and adoption of a variety of methods for sampling and identifying microplastics, there is now data showing the presence of microplastics in surface waters from all over the world. The difference between the methods, however, hampers comparisons, and to date, most studies are qualitative rather than quantitative. In order to allow for a quantitative comparison of microplastics abundance, it is crucial to understand the differences between sampling methods. Therefore, a manta trawl and an in situ filtering pump were compared during realistic, but controlled, field tests. Identical microplastic analyses of all replicates allowed the differences between the methods with respect to (1) precision, (2) concentrations, and (3) composition to be assessed. The results show that the pump gave higher accuracy with respect to volume than the trawl. The trawl, however, sampled higher concentrations, which appeared to be due to a more efficient sampling of particles on the sea surface microlayer, such as expanded polystyrene and air-filled microspheres. The trawl also sampled a higher volume, which decreased statistical counting uncertainties. A key finding in this study was that, regardless of sampling method, it is critical that a sufficiently high volume is sampled to provide enough particles for statistical evaluation. Due to the patchiness of this type of contaminant, our data indicate that a minimum of 26 particles per sample should be recorded to allow for concentration comparisons and to avoid false null values. The necessary amount of replicates to detect temporal or spatial differences is also discussed. For compositional differences and size distributions, even higher particle counts would be necessary. Quantitative measurements and comparisons would also require an unbiased approach towards both visual and spectroscopic identification. To facilitate the development of such methods, a visual protocol that can be further developed to fit different needs is introduced and discussed. Some of the challenges encountered while using FTIR microspectroscopic particle identification are also critically discussed in relation to specific compositions found.

**Electronic supplementary material:**

The online version of this article (10.1007/s11356-019-07274-5) contains supplementary material, which is available to authorized users.

## Introduction

Early plastic pollution research built on lessons from planktologists. For example, one of the metrics that have been used to report microplastic concentrations is the ratio of microplastics/plankton (Moore et al. 2005). Sampling was often done with surface water trawls, and identification was done visually with the aid of stereomicroscopy—methods that are still commonly used. Consequentially, a common cutoff size limit in many microplastic studies is 0.3 mm since that is also a common mesh size for studying zooplankton, fish eggs, larvae, and other organisms in the nekton.

It was, however, recognized early on that one method would not be adequate to assess microplastic pollution, particularly since microplastics come in many different shapes, sizes, and densities (Moore et al. 2005). This was further emphasized in a recent paper by Rochman et al. (2019). Additionally, a majority of plastic pollutants are expected to sediment (Woodall et al. 2014; Koelmans et al. 2017) or beach for periods of time. As the research field matured, other methods, specifically adapted to different situations and research questions, were developed (reviewed in Hidalgo-Ruz et al. 2012, Renner et al. 2017a, Prata et al. 2018). The different methods have allowed research to specifically identify microplastics in surface waters, biota, and sediments from all over the world. Several scientists have, however, noted the need to harmonize sampling methods (European Commission Joint Reseach Centre 2013; Lusher 2015; Van Cauwenberghe et al. 2015; Setälä et al. 2016; Rochman et al. 2017).

Today, surface waters remain an important area of study and particles above 0.3 mm are commonly sampled with trawls, such as the manta trawl, or filtering pumps. The degree of comparability between trawl and pump samples is however poorly understood. Previous studies using both trawl and pump methods with varying mesh sizes reported inconclusive results. A larger number of particles are quantified when using smaller mesh sizes, as shown for sea water samples taken by both a bongo net (mesh size 0.3 mm) and a submerged pump (mesh size 0.044 mm) resulting in 0.045 ± 0.093 and 2569 ± 1770 particles/m^3^, respectively (Cai et al. 2018). A sampling campaign in the Baltic, using both a submersible pump and a manta trawl (both with a mesh size 0.3 mm) on 10 different stations, resulted in a similar microlitter (including combustion particles) concentration range between 0.9 and 1.9 particles/m^3^ (Setälä et al. 2016). However, since there was only one replicate from each station, with low particle counts, a full statistical comparison was not possible, and there was no clear correlation with any category of microlitter for the two methods. Given the highly heterogeneous distribution of microplastics, a method comparison puts high demands on the study design. So far, information on the variation between replicates for different methods is lacking, due to the absence of replication in most studies. Hence, comparison between different methods is currently impossible.

Another challenge encountered when trying to compare results from microplastic surveys is the lack of comparable protocols for identification of microplastic, and other litter, in the samples. Identification is primarily done by visual identification, often with the aid of stereomicroscopy (Renner et al. 2017a). Of those that use visual identification for microplastics in environmental samples, half use it as a way to narrow down potential microplastics that are then further analyzed. The other half uses it as a stand-alone method (Renner et al. 2017a). Researchers use different protocols for distinguishing plastics from natural particles. For larger particles (> 1 mm), indicators include the absence of cellular or organic structures, and the presence of clear and homogenous colors, while fibers should be equally thick throughout their length (Hidalgo-Ruz et al. 2012). It has also been suggested that uneven crooked edges and/or distinctive colors can be used as characteristics to distinguish microplastics from naturally occurring particles (Viršek et al. 2016). The categories used for classification also vary but often refer to source, type, shape, color, and/or degradation stage (Hidalgo-Ruz et al. 2012). Of those studies that then move on to further characterize and analyze the particles, 28% use Fourier transform infrared (FTIR) spectroscopy (Renner et al. 2017a), where there are also discrepancies regarding how to obtain and interpret the spectra (Renner et al. 2017a, b, 2018). Guidelines for environmental monitoring protocols recommend spectroscopic analysis to complement visual identification, but there are no established recommendations on how the subset of particles should be selected (European Commission Joint Reseach Centre 2013; GESAMP 2019).

To be able to provide advice for future microplastics and microlitter sampling and analysis, in both research and monitoring, the aims were to (1) use field tests to examine differences between the most common methods for sea surface sampling (manta trawl and filtering pump), (2) use collected field data as the basis on which to discuss appropriate sampling volumes, and (3), in line with recent calls for harmonization (Hartmann et al. 2019), propose a comparable and less ambiguous protocol for identification and coupled visual and spectroscopic classification. This study has been focused on sea surface sampling, but many of the prerequisites and observations are equally valid for sampling microplastics in sediment and biota.

## Materials and methods

### Sampling

Sampling was performed in the Gullmar fjord on the Swedish west coast on October 10, 2017 (sampling center: 58°15,756 N 011°26,770 E), during a day with calm winds and little current. A nearby weather buoy was used to retrieve data on winds and currents before and during the sampling (station number 33025 ID Kristineberg, latitude: 58°15.41′N, longitude: 11°27.06′E).

### Trawl

**Fig. 1:**
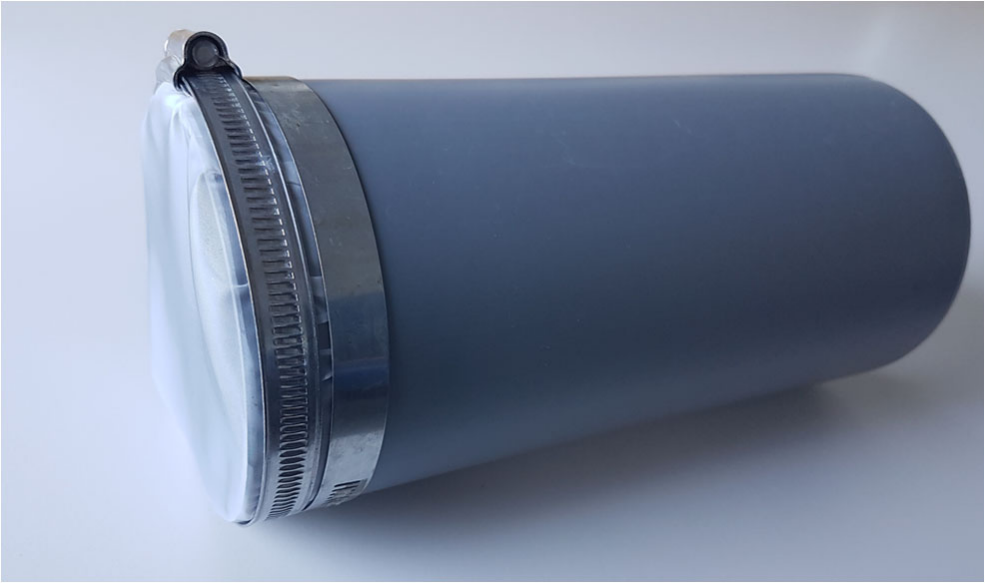
Detachable cod-ends, made from a polypropylene tube with a mesh attached to it, were prepared and cleaned in the lab to avoid contamination or loss of materials

The trawl consists of an aluminum frame with an opening width of 67.5 cm and height of 20 cm. A nylon net with a mesh size of 0.3 mm was attached to the frame. At the end of the net, detachable “cod-ends” were attached using hose clamps. The cod-ends were made from 3-mm-thick gray polypropylene tubes with a length of 23 cm and a diameter of 11 cm. Nets with the mesh size of 0.3 mm were attached to the tubes, also using hose clamps (Fig. 1). One tube was used per sample, and to avoid contamination, each tube was cleaned and prepared with Milli-Q (< 0.22 μm) in the lab, back-flushed, and sealed with aluminum foil before and after the sampling. Three different heights were marked on the sides of the trawl frame opening, to show a water height of 12.5 cm, 10.0 cm, and 7.5 cm, from the bottom of the trawl net level. To test the variation in water height, a miniature waterproof camera (GoPro) was attached. The water height in relation to the three marks was then recorded by converting the film to stacked images. The stacked images were analyzed in Fiji 64 for Windows, using a macro detailed in Online Resource 1a. The data were then analyzed based on times above, between and below the three different marks. Net size was analyzed by taking images with a Leica M205C camera stereomicroscope, and measuring differences in mesh width, length, and diagonal length.

Trawl samples were taken onboard RV Oscar von Sydow. The trawl was pulled alongside the ship from a boom for 0.5 nautical miles. The trawl was lifted as the ship turned, and was then pulled along the respective counter course to compensate for any differences due to currents. In total, ten samples were collected, and for each sample the course was altered by 72°. This created a cartwheel-shaped sampling pattern, in relation to which the pump was positioned at the center. The trawl repeatedly sampled across and back, Fig. 4). Each new trawl replicate started in five different directions (shifted by 36°) and then repeated again for a replicate size to ten. Three blanks were collected by preparing tubes and bringing them into the field, attaching them to the trawl and then analyzing in the same way as the samples in the lab. In addition, 2 carryover samples were taken after the first and last samples, by attaching clean code-ends and rinsing the trawl from the outside with seawater, to assess the carryover between samples.

### In situ filtration pump

**Fig. 2:**
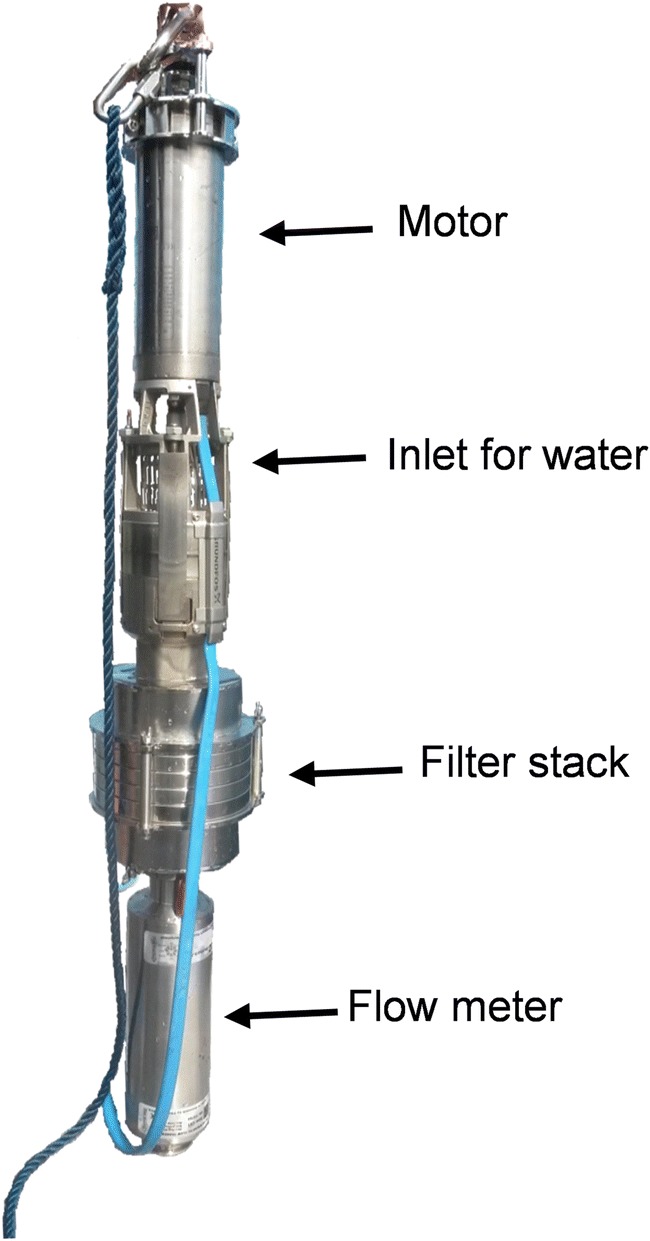
The pump consists of a motor above a water inlet grid (4 × 20 mm). The water is passed through a filter stack, and the filtered volume measured by an electromagnetic flowmeter. A synthetic rope is pictured here, but a stainless steel wire could also be used to further avoid risk of contamination

Six replicate pump samples were taken simultaneously with the trawl samples from a boat anchored in the center of the trawl trajectory. The filtration pump used is commercially available, and is the second generation developed by KC Denmark in the EU CleanSea project 2012–2014 (Fig. 2). The main differences from the first CleanSea pump relate to the motor and flowmeter. The current pump is fitted with a higher-capacity motor than its predecessor, and the original digital flowmeter has been replaced with an electromagnetic one. The pump is a stainless steel multi-stage Grundfos submersible pump. The 1-phase motor (0.55 kW/230 V, Grundfos MS402) is located at the top, followed by the water intake and a filter stack that fits three different filters. The filters are 14 cm in diameter and made in stainless steel using a laser cutter. Although this study focused on a 0.3-mm mesh size, it is possible to use sequential filtering with three different mesh sizes. The mesh size of the 0.3-mm filters was analyzed by taking images with an Axiocam ERc 5 s mounted on a light microscope (Stemi 508, Zeiss) and measuring differences in mesh width, length, and diagonal length. At the bottom of the pump, the electromagnetic flowmeter (PD340) measures the water volume passing the filters with an accuracy of (± 0.006% or 1.2 L at 20,000 L/h).

In this study, the pump was suspended horizontally about 0.5 m out from the boat. The 360° water intake, which measured approximately 20 cm in diameter, was situated 5–10 cm below the water surface. Prior to sampling, the filters were ultrasonicated, rinsed with deionized water, and stored in jars lined with aluminum foil. Two blank filters were processed and stored in the same way, except that they were not submersed underwater. Assuming a stainless steel wire is used for deploying the pump, the only plastic materials associated with the pump are the external electrical cables, a silicone rubber seal, and two internal black nitrile o-rings.

### Statistical evaluation

Within a given sampling area, there is a true concentration that can only be measured by sampling the entire area (the true population). As that is not practically possible, subsamples are taken to estimate the true concentration. A number of assumptions are then made in regard to the subsamples. These assumptions can for example be based on the assumption of Gaussian distributions (commonly referred to as a normal distribution). As microplastics typically arrive to the sampler as whole particles, i.e., discrete numbers, it is more suitable as a first approximation to adopt the assumption of a Poisson distribution, at least until the number of particles per analysis reaches high enough levels that the abundance (expressed as integers per sample) approaches a continuous Gaussian distribution, such that the Poisson statistical uncertainty is less than other measurement uncertainties. Based on the measured concentrations in the current study, these relationships are investigated as described below.

In order to calculate the probability value for a sample with the volume V (m^3^) of filtered water, one can use Eq. 1

1 $$ {\mu}_V=\lambda $$

where *λ* corresponds to the arithmetic mean of particles per sample. In Poisson distributions, the variance *σ*^2^ has been shown to be equal to the mean. Therefore, the standard deviation for that probability value can be calculated through Eq. 2

2 $$ {\sigma}_V=\surd {\upmu}_V $$

The probability value only approximates the true value at a high number of observations (which, in this case, can be obtained by a large sample volume). For a lower number of observations, Poisson statistics (e.g., Haight 1967) provide a probability density function *P*(*x* = *k*), as in Eq. 3:

3 $$ P\left(x=k\right)=\frac{\lambda^k{e}^{-\lambda }}{k!} $$

From the probability density function, the probability for different random results can be calculated based on a true mean and a set of observations.

If the distribution approximates a Gaussian distribution, the number of replicates necessary to detect a difference between samples can be calculated. For a *t* test between two samples, with a power of 80 (meaning that the difference would be detected 80% of the time), Eq. 4 can be used:

4 $$ N\approx 16{\left(\frac{\sigma }{D}\right)}^2 $$

where *N* is the number of replicates and *D* denotes the difference that should be detectable between samples.

### Visual analysis

All samples were kept in the cooling room at 5 °C between analyses. Visual analyses were performed using a stereomicroscope, following the MyVISPEC protocol outlined in Online Resource 2 and 3. One analyst analyzed all the samples to maximize comparability. Samples with a lot of seagrass and other macro debris were analyzed by carefully investigating both sides of the macro debris under the stereomicroscope and moving any adhered microlitter. All particles identified as anthropogenic were sorted into categories related to shape, solidity, color, longest length and perpendicular width, and descriptive categories (see MyVISPEC protocol in Online Resource 2 and 3). Each particle was assigned a particle ID and photographed using a Leica M205C camera microscope.

### FTIR analysis

FTIR analyses were performed using a Nicolet iN10, reflectance mode with an MCT detector cooled with liquid nitrogen. On each day of analysis, the inferometer was calibrated and a reference clear PE film was analyzed at the beginning and end of the day, as a quality control sample.

Although it is common to use a spectral resolution of 8 cm^−1^ (Renner et al. 2017a), a higher resolution of 4 cm^−1^ was employed for this work to provide (1) better discrimination between low-density polyethylene (LDPE) and high-density polyethylene (HDPE) (Gulmine et al. 2002), and (2) to distinguish characteristic peaks related to degradation, and increase the accuracy with which the automated library search functions. In this study, the spectra were analyzed with the software Omnic Picta (v9.1), using correlation with the software libraries Thermo Wizard library, Hummel polymer sample library, and Polymer laminate films. In addition, comparisons were made with two internal libraries, one with clean reference samples and one with household plastics and degraded plastics. All matches were confirmed with visual inspection of the key peaks for the respective polymer types.

## Results and discussion

### Pump

#### Precision

The pump filtered 20 m^3^ (± 1.2 L) per sample, as measured by the magnetic flow transmitter L, corresponding to a variation of 0.006%. Random measurements of the pump mesh size on three 0.3 mm filters (ten replicates for each filter) showed that the average width was 0.299 mm, with a standard deviation of 0.014 mm and values ranging from 0.279 to 0.328 mm. The diagonal length was on average 0.444 mm with a standard deviation of 0.016 mm.

#### Concentration

**Fig. 3:**
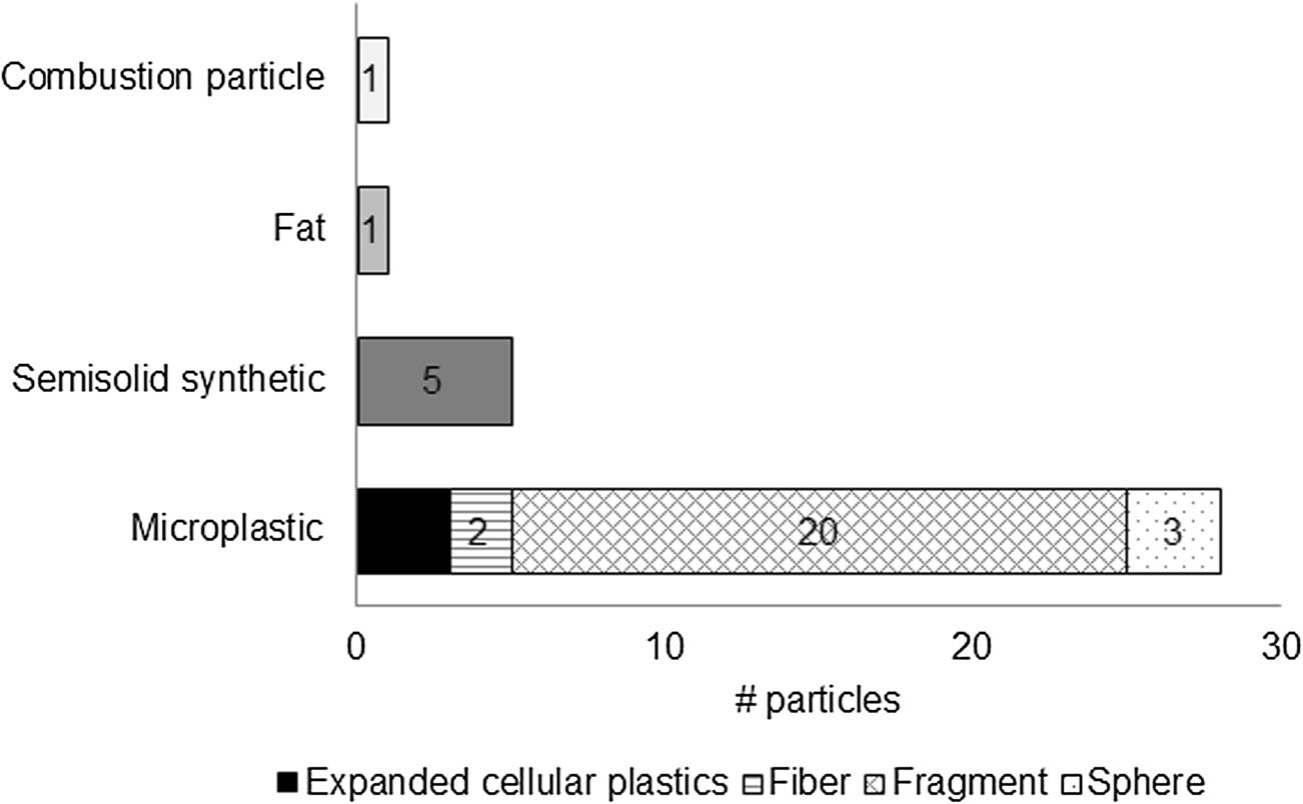
Composition of all particles found in the six pump samples

In the pump samples, between 0 and 13 particles identified as microplastics were found, a concentration that corresponds to 0–0.4 microplastics/m^3^. The highest concentration was found for the first replicate, and the lowest concentration for the last replicate. Additionally 0–2 other types of microlitter particles were found per sample as detailed under composition (Fig. 3). No microplastics were found on the blank filters.

#### Composition

In total, only 35 particles were identified as microlitter particles in the pump samples. Compositional differences between the replicates are therefore not reliable. In order to compare the compositions between the sampling devices, all particles found in the trawl and in the pump samples, respectively, were pooled for the evaluation. For the pump (Fig. 3), the majority (32/40) of the anthropogenic particles were visually identified as microplastics, while 5 were identified as semisolids, 1 as fat and 1 as a combustion particle. The microplastics were mainly fragments (20/32) but also had some expanded cellular plastics (3/32), some air-filled spheres 3/32), and a couple of fibers (2/32).

### Trawl

#### Precision

For the trawl, the video analysis resulted in a data set showing the times that the water height was between marks, or above or below the marks, in terms of images and corresponding time as detailed in Table 1.

**Table 1:** Height of the water in trawl in relation to the different marks

	Middle mark		Top mark		Low mark	
	Above	Below	Above	Below	Above	Below
Images	199	118	12	305	292	25
Time (s)	19.4	11.5	1.2	29.7	28.4	2.4

For the actual trawled distance, the ship nautical data rendering was used, which translated to an estimated average volume of 62 m^3^ per sample at the middle depth mark (Table 2). This estimate, however, depends if there is a deviation from the estimated trawl depth. A given trawling distance of 0.5 nautical miles and estimated water height position in line with the middle part of the trawl (10 cm) give a volume of 63 m^3^. On the other hand, if the trawl would be steady at the lower mark, the sampling volume would only be 47 m^3^, and conversely, with an assumed water level on the higher mark, the volume would be 78 m^3^, which would be a significant difference. Based on the video analysis, the water level was observed to be within the low and the top mark for the most part. If the results are extrapolated to a full trawl sample of 0.5 nautical miles, the volume would be 64 m^3^, 1 m^3^ more than the visually estimated volume. These are still quite crude measurements, but they indicate an error of at least 1% in volume estimations for the trawl.

**Table 2:** Comparison between pump and trawl

	Pump	Trawl
Volume per hour (m^3^)	25	> 140
Volume precision (%)	0.01	> 1
Microplastic concentration in comparative study (particles/m^3^)	0.23	0.51
Possibility to operate at varying depths	Yes	No
Possibility to operate with varying mesh sizes	Yes	No
Potential of plastic contamination from sampling device	Small	Medium

Random measurements on 10 spots in the mesh used for the trawl code-ends showed that although they were marketed with a specified mesh size of 0.300 mm, the average width was 0.258 mm, with a standard deviation of 0.01 mm and values ranging between 0.258 and 0.271 mm. The diagonal length was on average 0.379 mm with a standard deviation of 0.008 mm.

#### Concentration

The trawl had between 11 and 57 microplastic particles per sample, giving concentrations ranging between 0.18 and 0.92 microplastics/m^3^. Additionally, 5–26 other types of microlitter particles were found per sample.

Trawl blanks showed one paraffin particle. Therefore, contamination due to air exposure can be assumed negligible in these samples. Inter-sample contamination (extra trawl rinse) showed 1–3 particles per replicate, suggesting a potential carryover effect, although relatively small.

**Fig. 4:**
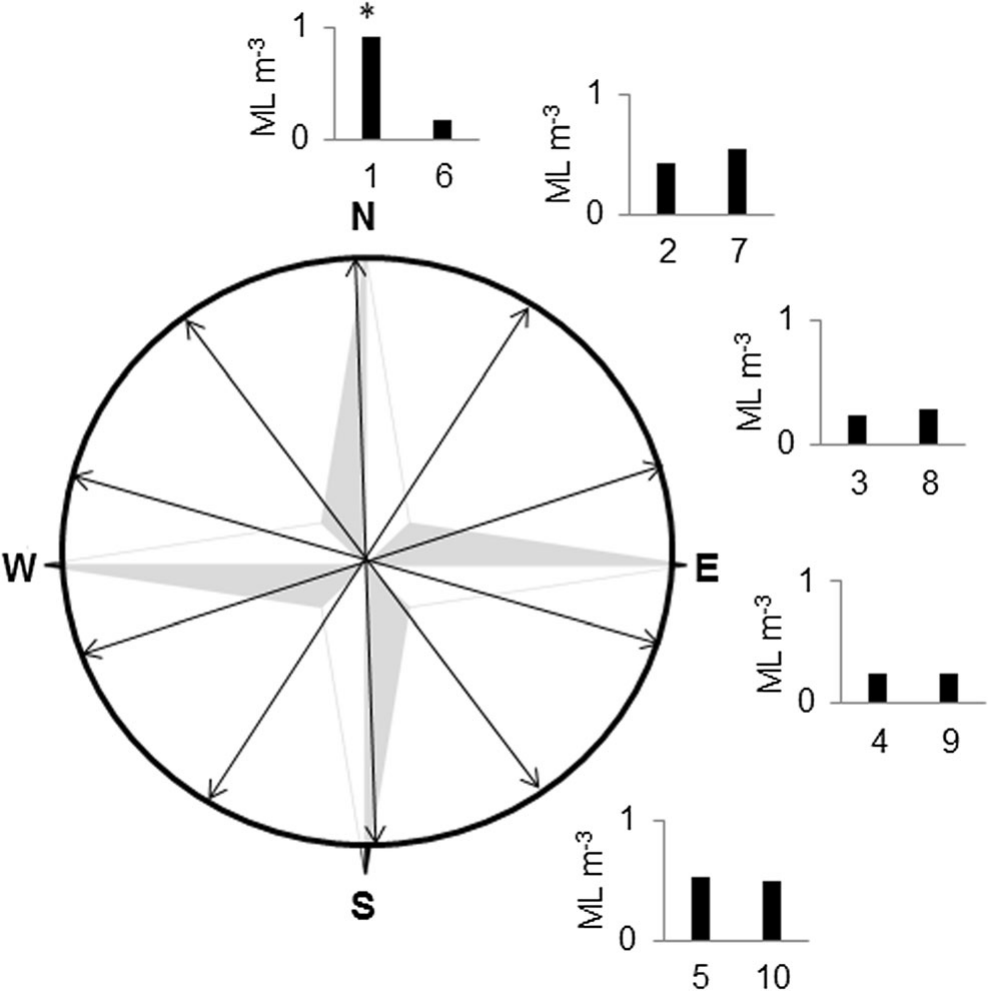
Concentrations of microlitter in trawl samples arranged according to trawl direction

Concentrations of microlitter in the trawl samples were observed to change throughout the day. When sorted according to trawl direction, it appeared that the difference between samples was more closely linked to trawling direction than to time of the day (Fig. 4). The samples that were sampled in the same direction had very similar concentrations, with the exception of sample 1 (denoted *), which was noted to pass through a more polluted streak (Online Resource 1b).

The small scale changes in current and wind direction, as measured by a nearby weather buoy, did not show any clear relationship to the sample volumes (Online Resource 1c). The currents during the day were between 3.6 and 9.6 cm/s, which could give a 5–16% variation in effective trawling speed dependent on the direction, but since all samples included a counter course haul, it should in principle be compensated for in the experimental design.

This also emphasizes the importance of including environmental parameters during sampling conditions.

#### Composition

**Fig. 5:**
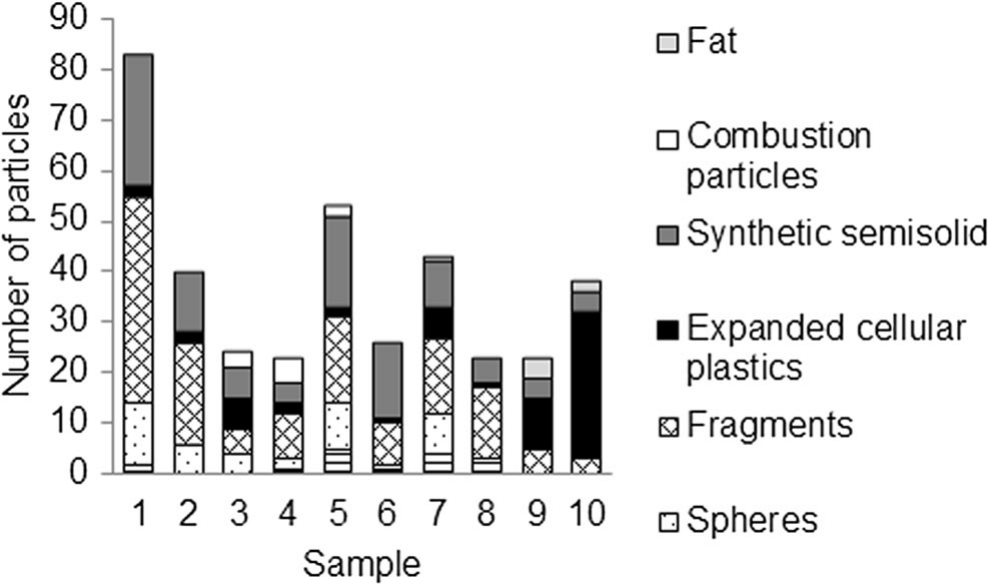
Compositional variation between replicates in the trawl samples

For the trawl, the number of particles per sample ranged between 23 and 83 microlitter particles. This is again insufficient for detailed compositional analysis between samples, but it can be used to illustrate the variation between replicates, as seen in Fig. 5. Among the first samples, the portion of fragments was higher than among the last samples, which were dominated by expanded cellular plastics.

**Fig. 6:**
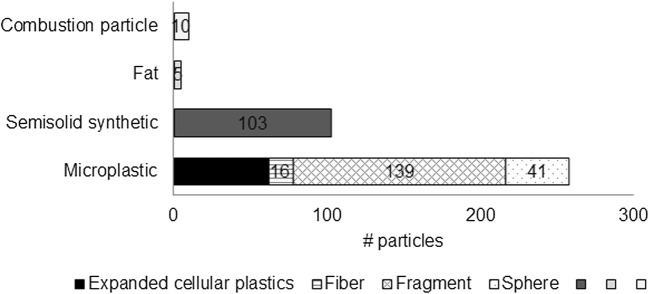
Sample composition in all ten trawl samples pooled

For the trawl 256/376, anthropogenic particles were visually identified as microplastics (Fig. 6). Other types of microlitter included occasional combustion particles (10/376), fat particles (5/376), and also semisolid synthetic particles (103/376), which made up a total of 20% of the total anthropogenic particles. Among the microplastics, the majority of the particles were classified as fragments (139/256), even when the fragments of expanded cellular plastics were excluded (62/256).

**Fig. 7:**
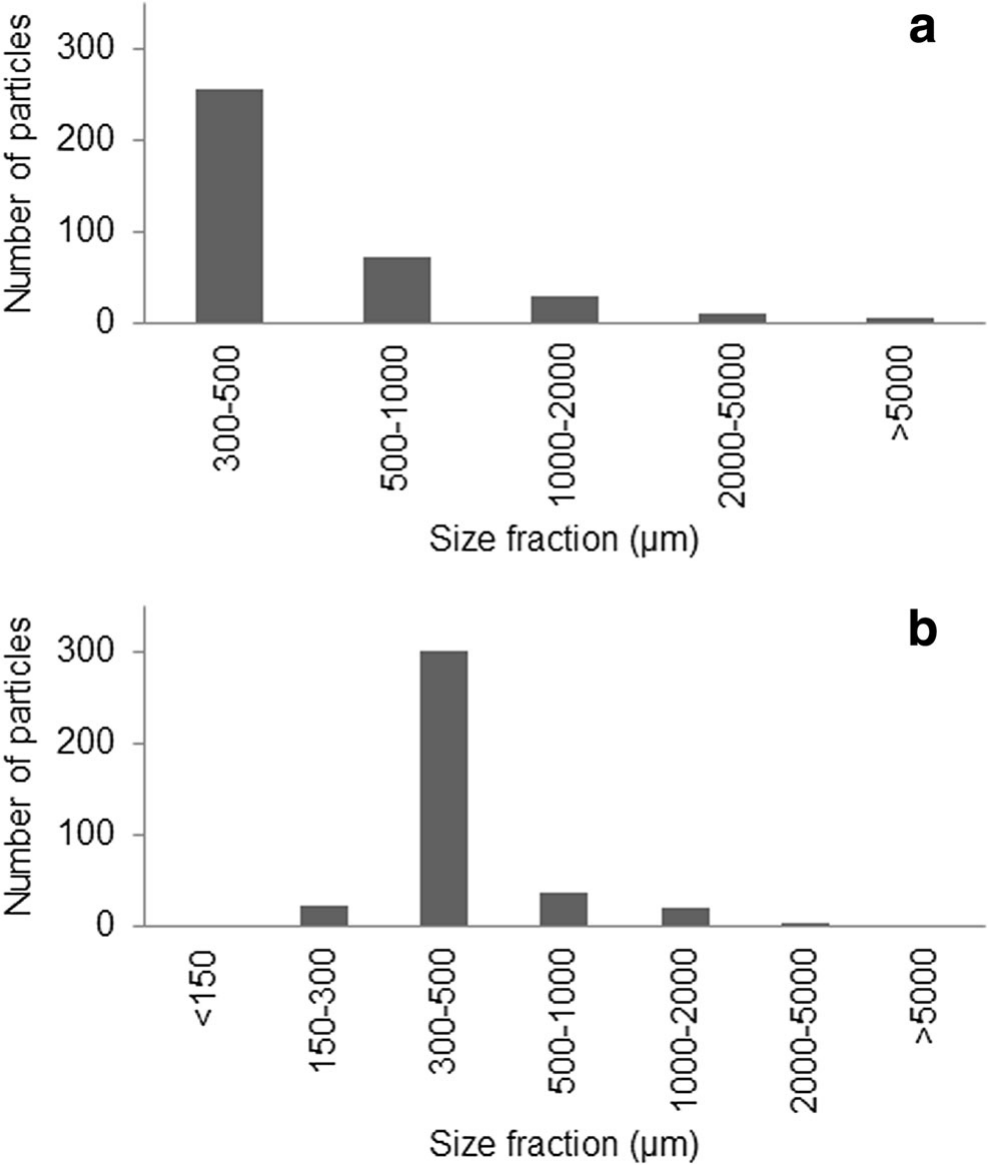
Length (**a**) and width (**b**) size distribution among microlitter particles in the trawl samples. The compositional difference between trawl replicates illustrates a potential temporal variation and the importance of replicates. The size distributions showed increasing numbers of particles in the lower sizes. The lower recorded numbers of larger particles also show that this type of sampling might not be suitable for particles above 2 or even 1 mm as only 48/376 particles were longer than 1 mm and 26/376 were wider. These results support recent recommendations regarding the definition of microplastics, where it has been suggested to use 1 mm as a cutoff rather than the commonly but arbitrarily adopted 5 mm cutoff limit (Hartmann et al. 2019). Consequently, a different approach (larger mesh and much larger sample volumes) would be required for 1–5-mm fractions.

**Fig. 8:**
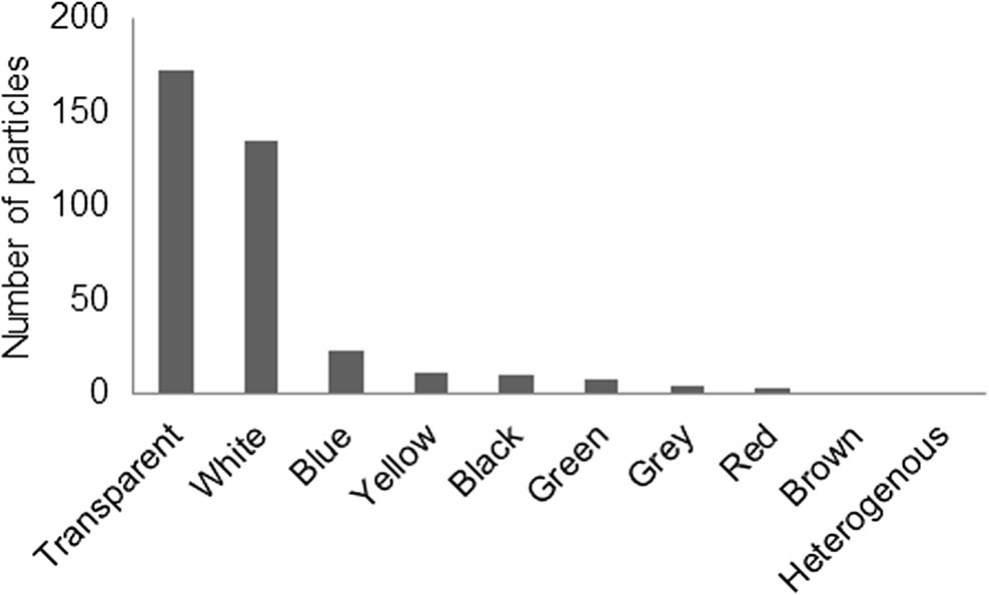
Color composition of microlitter particles in the trawl samples

The particles from the trawl were grouped according to approximate lengths and widths. The length was measured as the longest length, and the width was measured perpendicular to the length. The size grouping (Fig. 7) showed that the amount of particles increased with decreasing sizes down to 0.3 mm (the lower size limit). A few particles with a smaller width were recorded, but these were typically long fibers. Among all particles in the trawl, 82% were found to be transparent or white (Fig. 8).

### Statistical evaluation of the sampling results

**Fig. 9:**
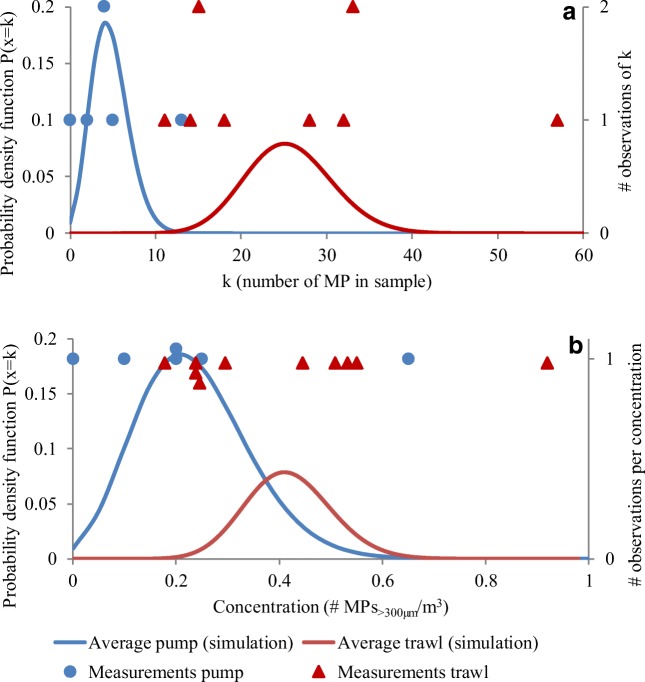
Modeled probability density function based on the measured data compared with actual observations of microplastics per sample for the pump and the trawl based on **a** number of particles per sample and **b** concentration of microplastics/m^3^

The modeled values and the observations (expressed as particles per sample and particles per volume) follow each other, although the trawl samples had more values towards the lower and the higher end of the modeled distribution (Fig. 9). The modeled values for the pump also illustrate the increased risk of false zero values with lower particle counts per sample since the curve cuts the *y*-axis. For the trawl, the curve is closer to a Gaussian distribution.

In order to calculate size distribution, morphology, or composition, an even higher number of particles would be desirable, for an acceptable level of Poisson relative to standard deviations. For large size fractions (> 300 μm) of microplastics and microlitter, this may not be practically feasible. Therefore, it is crucial that studies in this field do not draw conclusions beyond the inherent limitations. Analogously, here, from Eqs. 1 and 2 the inverse square root of the sample mean (26 particles) is taken, which would give 0.2 or a percentage of 20% relative standard deviation. Since that is less than the observed sample variability (here approximated to 55% by taking the sample mean through the Gaussian standard deviation), 26 particles could be considered enough for these types of samples. However, due to the high variations noted here, and the general patchiness that is associated with plastic pollutants, 26 particles per sample are towards the lower extreme, and should be considered a minimum.

In order to test how many replicates would be needed to allow for spatial or temporal comparisons, Power calculations according to Eq. 4 were performed. The results indicate that in order to distinguish between a sample with 26 particles and one with twice the amount, assuming a similar standard deviation to that observed here, at least 8 replicates would be necessary. If the minimum difference is instead raised to 50 particles/sample, only two replicates would be necessary. Conversely, if the same assumptions on distribution and standard deviation are used to examine a difference of 5 particles per sample, more than 200 replicates would be necessary to detect the difference 80% of the time.

The acceptable variation and standard deviation will ultimately be a balance between the research questions and what is economically and practically feasible. It is however vital to recognize the limitations imposed by low particle counts and replicate numbers for under-sampled fractions (especially larger sizes and uncommon microlitter types) in order to avoid drawing false conclusions not supported by the obtained data.

### Comparison between sampling methods

The repeatability of the volume measurements was notably higher for the pump. The precision of the trawl was found to primarily depend on the ability to keep it leveled in the water and visually estimate the water height during sampling. Although our tests using a waterproof camera indicated that the estimates used in this study might be 1 m^3^ higher than actual trawled volumes, it should be noted that the height has been observed to vary more in rougher weather conditions, which would increase the uncertainty in the volume estimation. The actual trawled volume will also depend on the current and wind conditions during sampling, which might also to some extent explain the differences in concentration associated with trawling direction. The uncertainty in volume is probably the biggest uncertainty in sampling with a trawl, and there is room for innovative approaches to measuring it more precisely.

The accuracy of the mesh size is likely to depend on the supplier, but should be recorded prior to sampling. Moreover, due to the variations and accuracy level in mesh size, it is more appropriate to report the sizes as > 0.3 mm than the conventional 333 μm.

Sampled concentrations of microplastics and other microlitter differed between the two methods. The trawl sampled higher concentrations than the pump. The pump also sampled a lower volume and, consequently, had lower number of particles per sample. Statistical evaluations showed that the low number of particles gave rise to increased variability when recalculated to concentration. Low particle counts per sample also resulted in higher probability of obtaining a false zero value. However, variations in statistical uncertainty between the methods are mainly due to differences in sampling volume, and can therefore be overcome by increasing the sampling volume, or sampling in areas with higher levels of contamination. Our results indicate that a minimum of 26 particles per sample is necessary, but determining appropriate sample sizes is not straightforward and will depend on the research question. Geelhoed and Glass examined theoretical ways of determining the minimum sample size based on the relationship between the sample size and the sample variation. In their review, they found that none of the existing theories met all the necessary criteria (Geelhoed and Glass 2004). The relationships are therefore complex, but it is nonetheless clear from the current study and relevant literature that future microplastics studies need to consider (1) the sample volume; to get a representative amount of particles per sample and (2) replication; to accommodate for the plastic pollution patchiness. The latter becomes particularly important for stationary sampling devices such as filtering pumps compared with trawls that cover a larger sampling area and therefore are less affected by the patchiness of the pollutants. Concentrations in surface water studies are known to differ over several orders of magnitude. If the results here are recalculated to microplastics/km^2^, the average levels (41,000 microplastics/km^2^) are similar to concentrations found in accumulation zones according to a global inventory of microplastics carried out over several years (Van Sebille et al. 2015). Higher concentrations have been reported in surface waters closer to urban areas (Mani et al. 2015; Zhang et al. 2017; Hassellöv et al. 2018). Aside from urban waters, our results are therefore comparable with commonly sampled areas in terms of particles per volume. The volumes used for the pump sampling may therefore be more suitable for samples taken closer to cities or more polluted areas. It should also be noted that an important merit of the filtration pump is its capability to sample lower sizes in cascade filtration setup, which manta trawls cannot.

The differences in concentrations between the trawl and the pump in the current study were mainly attributed to the compositional differences between the methods, although the low particle count in the pump sample did not allow for any in-depth analysis of the compositional difference. The two methods sample at slightly different depths. The trawl samples the full surface microlayer, wherefore the trawl might be more efficient at sampling microplastics that float on top of the surface, such as expanded cellular plastics. The trawl also had a higher amount of microplastic spheres (62/256) with entrapped gas, sometimes called micro-balloons. The pump has its sample intake at 5–10 cm below the surface wherefore it might not be as efficient in sampling the sea surface microlayer, where Song et al. (2014) showed that lighter microplastics tend to accumulate (Song et al. 2014). The difference in sampling depth might also explain the higher amount of spheres found in the trawl, as the spheres were often filled with air. Since the majority of the microplastics were classified as fragments (84% when fibers and expanded cellular plastics were included), it is likely that the greatest source of microplastics in this area is fragmentation of larger items, which has also been noted in previous studies (Hassellöv et al. 2018; Karlsson et al. 2019). The spheres were not visually weathered and seemed to be intact primary plastics. The composition results are based on insufficient sample sizes for extrapolation, but give a snapshot image of the contamination at the time of the sampling in this area.

### Visual analysis

Most studies on microplastics in the sea surface have used visual identification and sorting of the particles (Hidalgo-Ruz et al. 2012). Intra- and inter-lab comparisons showed that different analysts get different results. Therefore, one analyst did all the analyses in this study. As tactile parameters were included to distinguish between solid and semisolid particles, no false positives were identified with the FTIR analysis, which also indicates that careful visual analysis performed by experienced analysts may be suitable for samples down to 0.3 mm. The risk of underestimation should however not be overlooked, as previously illustrated (Karlsson et al. 2016). The authors therefore encourage a combination of visual, tactile, and spectroscopic methods to rule out false positives, and to help avoid false negatives, such as the spheres which could be mistaken for air bubbles, glass spheres (Karlsson et al. 2019), or possibly eggs. The risk of false negatives and positives increases with smaller sizes (Primpke et al. 2017), and it is therefore appropriate that JRC Guidelines (European Commission Joint Reseach Centre 2013) recommend that visual identification should not be done without complementary identification techniques for samples below 0.1 mm. The same guidelines state that most of the microlitter consists of microplastics, an assertion corroborated by the present study. However, the high percentage of other types of microlitter (> 20%) warrants their inclusion in future surveys/monitoring programs. Through including the identification of particles such as paraffin, false positives are also avoided.

Categories used for sorting particles typically vary between analysts (Hidalgo-Ruz et al. 2012) but often require the analyst to make assumptions about the type or source of the particles, based on visual identification alone. These subjective assumptions can increase the risk of drawing false conclusions; for example, expanded particles can be several polymer compositions and a weathered piece of colored polyethylene may visually be similar to a paint flake. Additionally, this type of protocol requires a highly experienced analyst to visually recognize different particles. The importance of experience and training cannot be understated in these types of analysis, but in order to increase objectivity, a protocol based on a set of subcategories related to particle morphology (such as shape, color, solidity, and size) is suggested (Online Resource 2 and 3). These subcategories can then be coupled with spectroscopic composition identification. In accordance with the recent article on definitions of microplastics (Hartmann et al. 2019), it is suggested that particle composition and potential sources are only stated when they can be conclusively determined. Therefore, the term “filament” to describe fibers that appear to be fragments of long filaments is avoided, and instead the all-encompassing term “fiber” is used.

A section on color is also included in the protocol, since it can facilitate visual separation, and a potential bias towards more bright colors cannot be ruled out (Hidalgo-Ruz and Thiel 2013).

Initially, the protocol differed between foils and other types of fragments, but as these were sometimes ambiguously identified, it was decided to group these together and use a more neutral differentiation between them, based on the tactile section as either rigid or pliable (Online Resource 2). This section also assisted in distinguishing between particles that can and cannot be quantified. Pliable particles and fibers with a width smaller than the mesh size cannot be assumed to have been reliably quantified by these sampling methods, since they can slip through the mesh and their presence is therefore dependent on several factors such as clogging and entanglement. Representative examples of the different types of microlitter are shown in Online Resource 2.

Although the use of the more objective protocol turned out to be slightly more time consuming, it provided a less ambiguous way of categorizing the particles (moving away from qualified guessing). It also provided more detailed data on the particle composition and proved to be a practical way of separating the particles. It is therefore recommended that future studies on microplastics or microlitter include a detailed description of identification parameters. The suggested protocol, MyVISPEC, can be found in Online Resource 3 and is free to use and alter for related purposes.

### Fourier transform infrared spectroscopy analysis

Similar to visual analysis, there are few protocols for analyzing environmental plastics with FTIR spectroscopy and it has been emphasized that the parameters for acquisition and especially spectral identification are rarely specified in scientific articles (Renner et al. 2017a). Environmental transformations of plastics (such as oxidative weathering, hydrolysis, biofilm formation) give rise to new functional groups, chain scissoring, and crystal state changes. Consequently, spectral characteristics change, in that peaks decrease and broaden, and new distinct peaks appear, while broad regions characteristic of –OH and –NH groups appear as a result of biofilm formation (Gulmine et al. 2003; Jakubowicz et al. 2006; Karlsson et al. 2018). Manual scrutiny of automatic spectral identification, even though it is more time-consuming, gives a more accurate identification of weathered microplastics (Renner et al. 2017a). Additives can also change spectra to some degree. Identification of unknown particles is therefore not always (or rarely) possible with pristine polymer library matching, but requires both environmental plastic reference libraries and some expert judgment, to scrutinize the computer matching. Sometimes, the authors state that they have used a matching or hit quality index cutoff limit of 60% or 80%, but without specifying which software was used (e.g., Yang et al. 2015) or which library (e.g., (Woodall et al. 2014; Avio et al. 2015; Yu et al. 2016), or which criteria/settings (e.g., (Woodall et al. 2014; Yang et al. 2015; Avio et al. 2015; Castillo et al. 2016). In such cases, a matching rate cutoff is in fact rather nonspecific. Microplastics analysis with FTIR requires knowledge of polymer spectroscopy (Song et al. 2015; Mecozzi et al. 2016) and can be aided by better-adapted preprocessing algorithms and analytical algorithms, tailored to environmentally weathered materials. As developed, for example, by Renner et al., they managed to increase the accuracy of identification from 76 to 96% compared with conventional library searches, by limiting the comparison to regions with vibrational bands and applying new search algorithms (Renner et al. 2017b). Even with the improved identification rate, Renner and colleagues still also recommend visually double-checking the spectra after the automatic recognition.

**Fig. 10:**
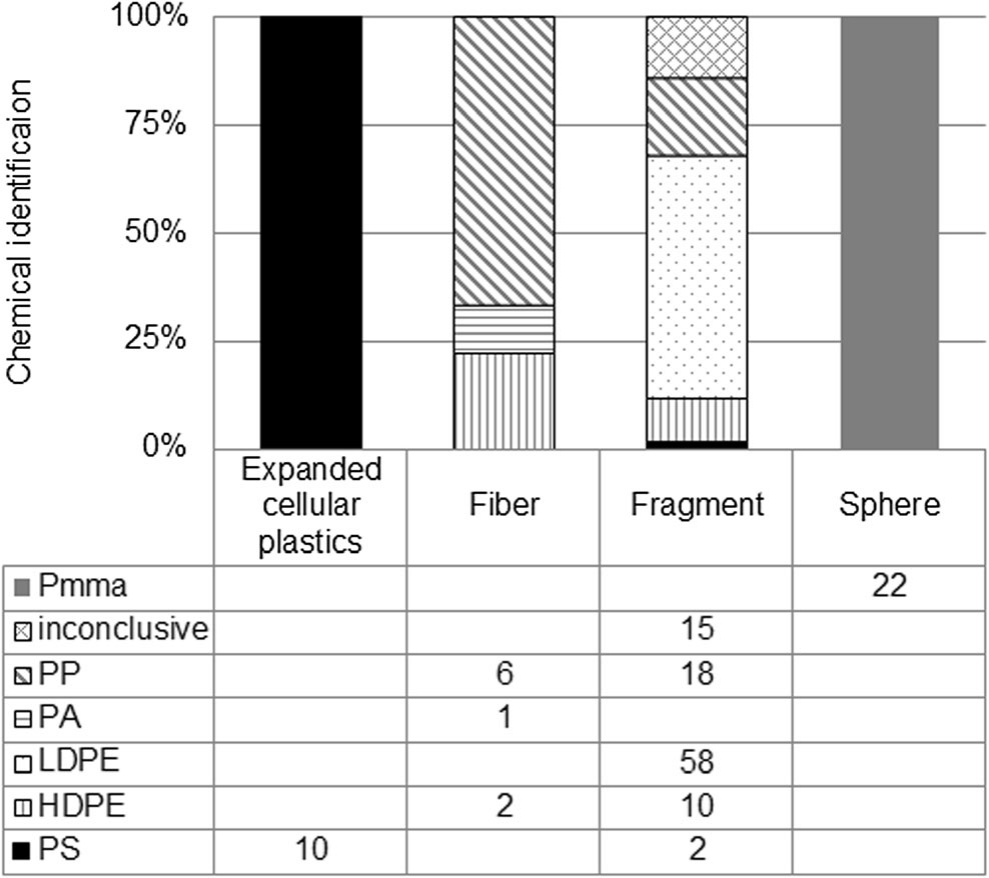
FTIR analysis of 144 particles from the pump and the trawl

As there were not enough particles among the pump samples to compare chemical compositions between the two sampling methods, the total amount of particles was pooled for chemical analysis. All particles that were visually identified as microplastics in the pump samples, and in four random trawl samples, were analyzed. In total, 144 suspected microplastics were analyzed corresponding to 50% of the total 286 microplastic particles (Fig. 10). The analyses identified all of the analyzed expanded cellular plastics (10) as polystyrene (PS) and all spheres (22) as polymethylmethacrylate (PMMA). Fibers were either polypropylene (PP) polyamide (PA) or high-density polyethylene (HDPE). Fragments were identified mainly as low-density polyethylene (LDPE), polypropylene (PP), or HDPE. Two fragments were also identified as PS. Of the fragments, 15 spectra were of insufficient quality for reliable identification the composition.

Additionally, 64 of the semisolid synthetic particles were analyzed, corresponding to 59% of the total. Seventy-seven percent were identified as paraffin due to their similarities with reference spectra of paraffin, PE, and PP. Twenty-three of the particles did not produce spectra of sufficient quality for identification.

**Fig. 11:**
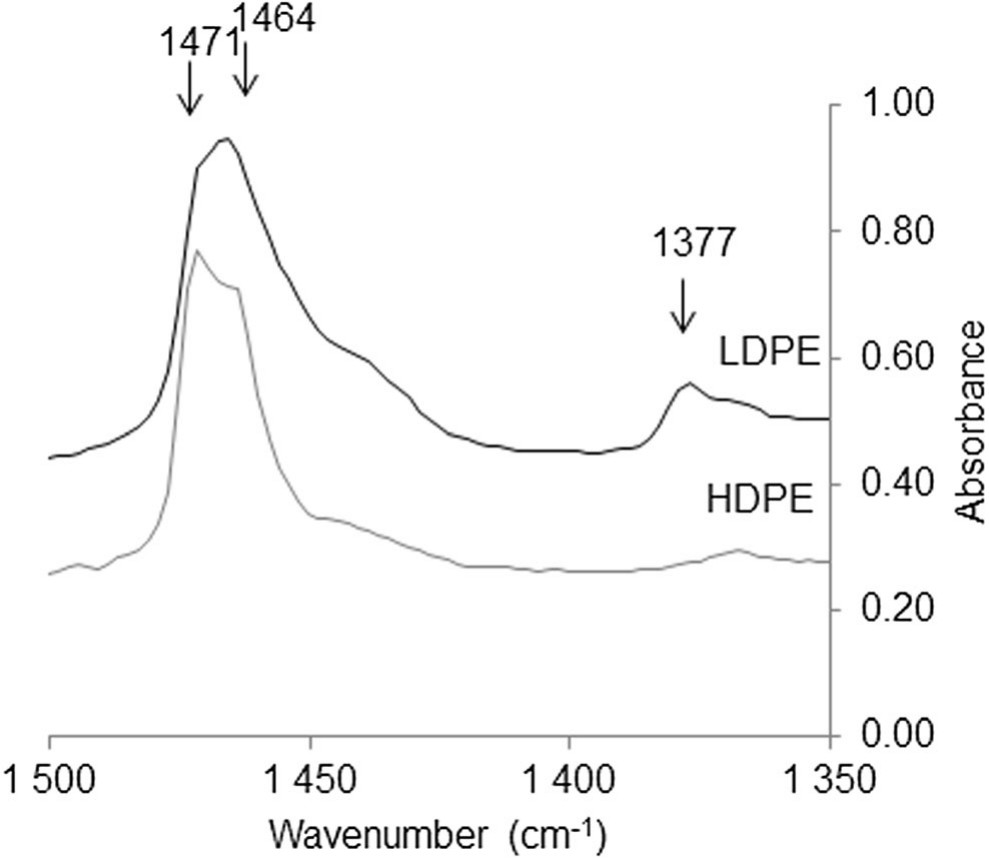
To distinguish between LDPE and HDPE, the peak at 1377 cm^−1^ is often used. Additionally, the peaks at 1471 and 1464 cm^−1^ can give some indication as these indicate the CH_3_ and CH_2_ groups, respectively

Another benefit of visual inspection of the spectra is that it allows for further interpretation. One example is the distinction between LDPE and HDPE which is typically done through looking at the presence or absence of a peak at 1377 cm^−1^ (Gulmine et al. 2002). This difference would not be noted by a typical library search. HDPE and LDPE also differ from each other in the relative height of the peaks at 1471 cm^−1^ and 1464 cm^−1^ (Fig. 11). In this study, 83% of the PE was identified as LDPE and 17% as HDPE.

Microplastic studies sometimes conclude that some particles could not be identified as plastics by FTIR, and that they therefore are not plastics—without stating the identity of those particles. This risks giving the false impression that the visual identification was wrong, even though it could in fact be due to a false negative FTIR identification. Here, we have instead chosen to write “inconclusive” if we cannot make a positive identification with FTIR. No false positives were identified with the FTIR analysis. Microspheres have proved challenging to visually differentiate from glass microspheres, and FTIR identification facilitated recognizing visual characteristics (Karlsson et al. 2019). If only visual studies are available, the microspheres can be tested with acetone, which would have a strong effect on PMMA, but not on glass.

Paraffin also proved challenging to identify using only visual or spectroscopic tests. The paraffin polymer can generate false positives as it shares several similarities with PE and PP, both visually and spectroscopically. As paraffin has shorter chains, some differences in the spectra can be noted in the ratio between 1464 and 1471 cm^−1^, but this difference was not always clear. There are also some indications that the CH_2_ band around 2902 cm^−1^ can be helpful in separating paraffin from PE and PP, although this relationship needs to be verified with several types of paraffin.

## Conclusions

The results presented here illustrate some of the challenges associated with sampling microplastics. A visual identification protocol is presented to encourage more objective sample analysis. The high prevalence of paraffin and its visual and spectroscopic similarity with polyethylene and polypropylene show the importance of including tactile probing during identification. It also exemplifies why smaller fractions would not be possible to quantify without supplementary chemical techniques, such as vibrational spectroscopy, and adds an important argument as to why visual identification and FTIR can be used as complementary techniques for microplastics identification. For subsequent analysis with FTIR, the results obtained here further illustrate the many uncertainties and pitfalls potentially encountered when using an automated library search. Therefore, a high level of caution is recommended. Due to the patchy nature and complexity of these contaminants, high sample volumes and replicates are necessary to provide quantitative and compositional data. In comparing the two sampling devices, it was found that the pump was more accurate in volume measurement and versatile for point sampling and filter size choice. Due to the lower sampling volume, it might be most suitable for sampling in areas with higher level of contamination. The trawl on the other hand integrates samples over a larger area and therefore helps circumvent some of the problems related to patchiness. It was also more efficient in sampling the sea surface microlayer and therefore resulted in higher concentrations and more microspheres and expanded cellular plastics than the pump. Regardless of sampling method, the tests showed that it is important to ensure that a high enough volume is sampled to obtain a sufficient particle count. It was concluded here that a minimum of 26 particles per sample was needed to allow for a quantitative comparison, although several replicates would be necessary to detect differences between the location sampled here and a hypothetical location with double the microlitter concentration. For compositional comparisons, even higher particle numbers would be necessary.

## Electronic supplementary material

ESM 1 (DOCX 233 kb).

ESM 2 (DOCX 3658 kb).

ESM 3 (XLSX 23 kb).
