# Supplementary material for: Comparison between manta trawl and in situ pump filtration methods, and guidance for visual identification of microplastics in surface waters
Source: Environ Sci Pollut Res Int. 2019 Dec 18;27(5):5559–71. doi: 10.1007/s11356-019-07274-5 (PMC7028838; doi:10.1007/s11356-019-07274-5)
Supplement: Supplementary file 1 — (DOCX 233 kb). [file 11356_2019_7274_MOESM1_ESM.docx]

**Comparison between manta trawl and in situ pump filtration methods and guidance for visual identification of microplastics in surface waters.**

*Environmental science and pollution research*

Therese M. Karlsson^1^, Anna Kärrman^2^, Anna rotander^2^ & Martin Hassellöv^1^

^1^ Department of Marine Sciences, University of Gothenburg, Kristineberg 566, Fiskebäckskil 45178, Sweden

^2^ MTM Research Centre, School of Science and Technology, Örebro University, Sweden

Corresponding author

Martin Hassellöv

[Martin.hassellov@gu.se](mailto:Martin.hassellov@gu.se)

1. Macro used for image analysis of the trawl video

run("Images to Stack", "name=Stackofall title=[] use");

run("Duplicate...", "duplicate");

makeRectangle(4, 59, 46, 261);

run("Crop");

//makeLine(6, 120, 8, 158);

//run("Measure")

//run("Set Scale...", "known=5 unit=cm");

run("8-bit");

setAutoThreshold("Minimum dark");

run("Convert to Mask", "method=Minimum background=Dark calculate black");

//setTool("rectangle");

for (s = 1; s < nSlices(); s++){

setSlice(s);

run("Measure");

}

b)


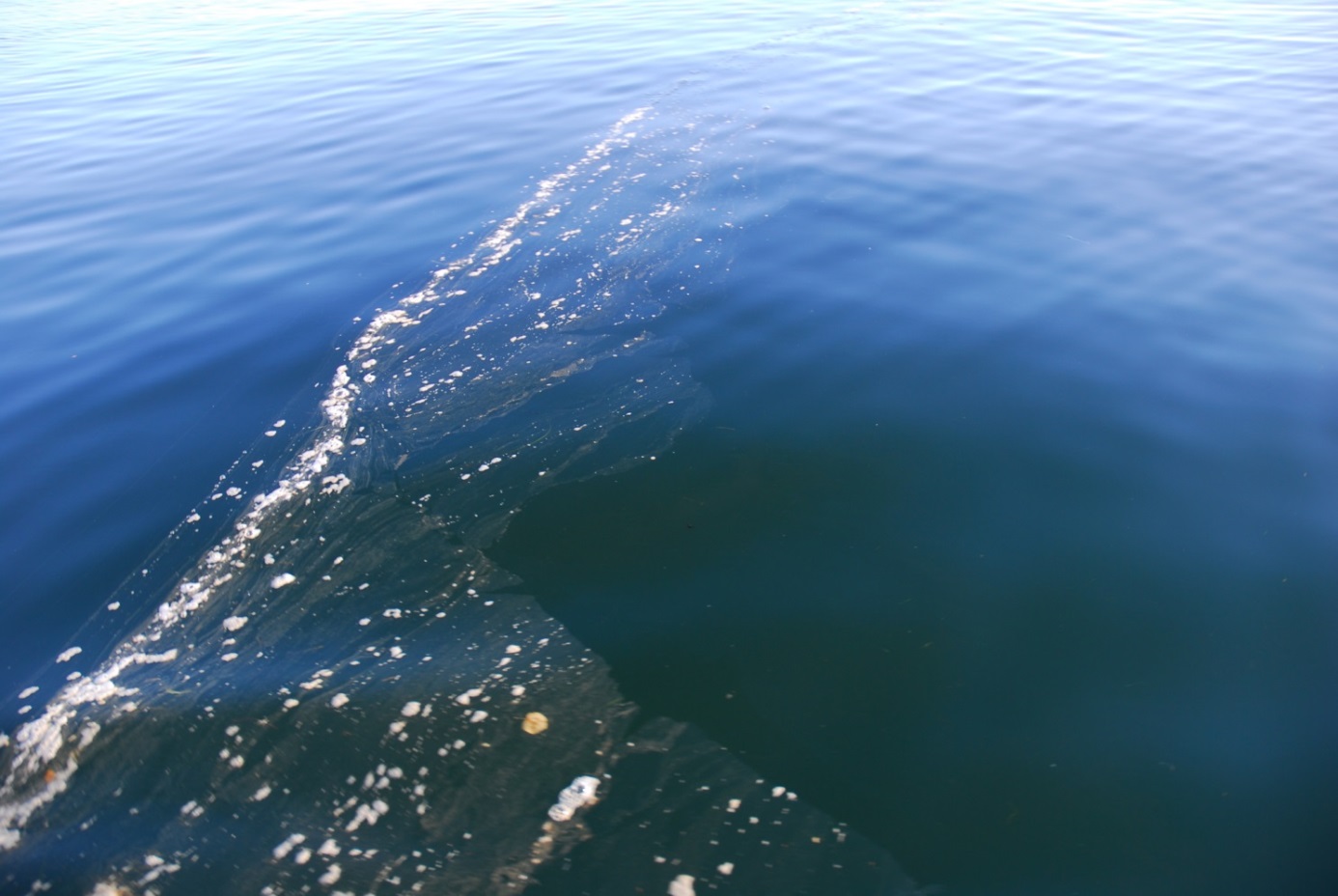


Polluted streak that the first trawl sample passed through

c) Weatherdata on sampling day as recorded by a nearby weatherbuoy

| **Time** | **Wind direction (°)** | **Wind Speed (m/s)** | **Air temperature (°C)** | **Water temperature (°C)** | **Salinity (psu)** | **Current direction 1 m (°)** | **Current speed 1 m (cm/s)** |
| --- | --- | --- | --- | --- | --- | --- | --- |
| 0 | 85 | 1.6 | 5.4 | 11.68 | 25.79 | 189.5 | 0.6 |
| 100 | 126 | 1.4 | 4.7 | 11.79 | 25.7 | 238.6 | 6.9 |
| 200 | 155 | 1.6 | 3.3 | 12.03 | 26.41 | 239 | 9.3 |
| 300 | 119 | 0.8 | 3 | 12.22 | 26.18 | 209.3 | 8.4 |
| 400 | 128 | 1 | 3.1 | 12.5 | 26.17 | 198.4 | 9.5 |
| 500 | 99 | 1.7 | 2.5 | 11.22 | 25.48 | 292.4 | 1.8 |
| 600 | 112 | 1.6 | 2.3 | 11.77 | 26.12 | 268.5 | 3.8 |
| 700 | 92 | 1.6 | 4.1 | 11.94 | 26.49 | 218 | 4.1 |
| 800 | 64 | 0.9 | 5.7 | 13.23 | 28.06 | 257.5 | 0.9 |
| 900 | 284 | 0.3 | 8 | 12.49 | 26.93 | 237.3 | 1.7 |
| 1000 | 287 | 0.8 | 12.6 | 13.67 | 29.08 | 220.4 | 9.6 |
| 1100 | 262 | 1.6 | 8.6 | 12.5 | 27.69 | 182.3 | 4.9 |
| 1200 | 217 | 0.9 | 9.1 | 13.54 | 28.86 | 225 | 5.4 |
| 1300 | 210 | 2.3 | 9.1 | 13.68 | 29 | 24.4 | 3.6 |
| 1400 | 157 | 3.2 | 8.5 | 13.35 | 28.22 | 195.9 | 4.4 |
| 1500 | 130 | 3.2 | 8.1 | 13.78 | 29.2 | 238.4 | 6.1 |
| 1600 | 121 | 2.7 | 7.1 | 14.91 | 31.67 | 310.9 | 6.3 |
| 1700 | 145 | 1.6 | 7.1 | 14.6 | 31.11 | 261.1 | 6.5 |
| 1800 | 207 | 1.6 | 7.1 | 13.49 | 29.11 | 230.6 | 7.7 |
| 1900 | 179 | 2.7 | 7 | 13.04 | 28.49 | 223.4 | 2.5 |
| 2000 | 101 | 2 | 7.3 | 13.03 | 28.74 | 150.7 | 4.7 |
| 2100 | 58 | 4.7 | 7.6 | 14.26 | 30.7 | 162 | 8.6 |
| 2200 | 50 | 5.1 | 7.3 | 14.67 | 31.72 | 174.8 | 4.4 |
| 2300 | 327 | 3.8 | 7 | 13.87 | 30.33 | 151 | 9 |
