# Supplementary material for: Comparison between manta trawl and in situ pump filtration methods, and guidance for visual identification of microplastics in surface waters
Source: Environ Sci Pollut Res Int. 2019 Dec 18;27(5):5559–71. doi: 10.1007/s11356-019-07274-5 (PMC7028838; doi:10.1007/s11356-019-07274-5)
Supplement: Supplementary file 2 — (DOCX 3658 kb). [file 11356_2019_7274_MOESM2_ESM.docx]

**Comparison between manta trawl and in situ pump filtration methods and guidance for visual identification of microplastics in surface waters.**

*Environmental science and pollution research*

Therese M. Karlsson^1^, Anna Kärrman^2^, Anna Rotander^2^ & Martin Hassellöv^1^

^1^ Department of Marine Sciences, University of Gothenburg, Kristineberg 566, Fiskebäckskil 45178, Sweden

^2^ MTM Research Centre, School of Science and Technology, Örebro University, Sweden

Corresponding author

Martin Hassellöv

[Martin.hassellov@gu.se](mailto:Martin.hassellov@gu.se)

# Manual for the identification and categorization of microplastics >300 µm

## General

The purpose of this manual is to provide an introduction to visual analysis of microplastics >300 µm and to present a framework that can be further adapted and harmonized for future microplastic research and monitoring.

Before analysing the particles, ensure that the sample volume is big enough and consider using an extraction technique (reviewed in GESAMP 2019) if the samples have a lot of organic materials. Once you start analysing make sure that you have good light sources with lights with an adjustable angle, good tweezers and a needle. During analysis, look for cell structures which indicate that it is not a synthetic material. In lower magnifications this may look like the particle is “sparkly”, if so you can use a higher magnification to check for cells. It is also good to look at the breaking point and scratches on the surface as they are often distinct from non-plastic particles.

While sorting it can be useful to make small water drops in the Petri dish and use a small needle to transfer the smaller particles. Dip the needle in the water and then gently touch the particle so that it sticks. Then put it in a water drop in a multiwell dish. The water makes the particle adhere better – otherwise the static electricity will make the particles fly around. Be aware that some analytical methods such as FTIR are sensitive to water; the particles may therefore need to dry again before further analysis. Once the particles are sorted they can be assigned individual particle IDs and descriptors regarding shape, size and colour can be filled out in the protocol as shown in the workflow below (Figure 1).


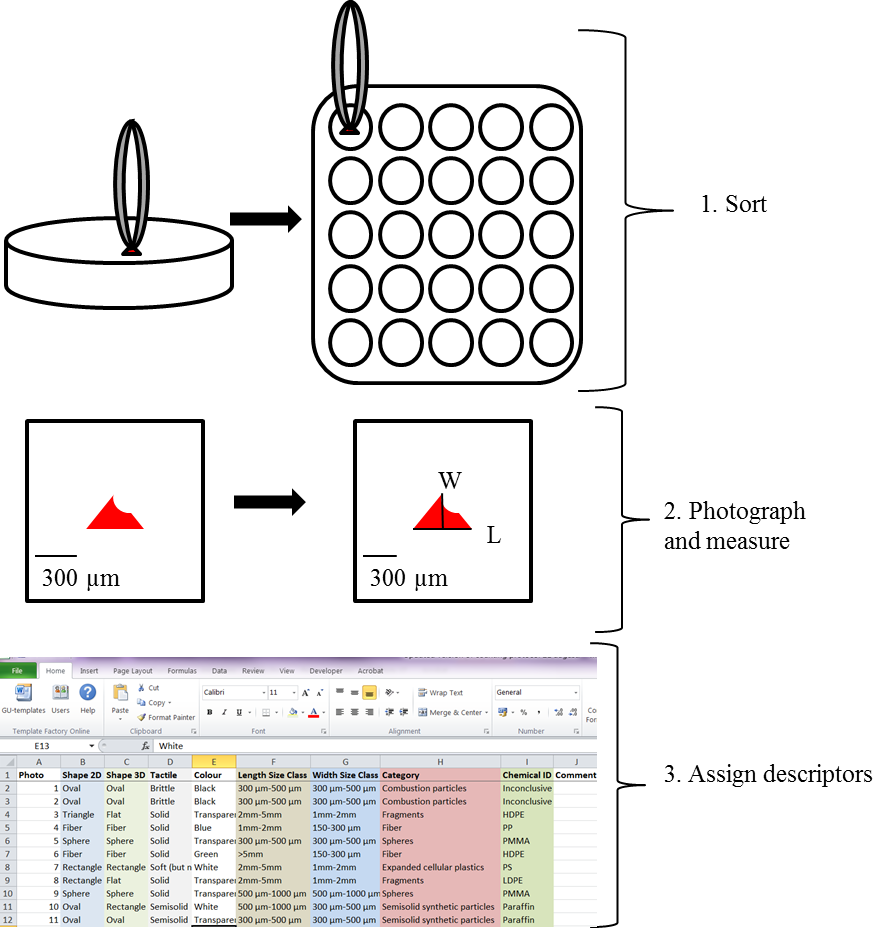


**Figure 1**. Suggested workflow for particle categorization.

# Protocol

## Sheet 1- Sample information

The sample information sheet provides an overview of the sample background and helps the analyst to keep track of the analyses that have been done (Table 1).

**Table 1.** Parameters in sheet 1 of the protocol used to provide an overview of the background information of the sample.

| Sample ID |  |
| --- | --- |
|  |  |
| **Sample info** | Sample date |
|  | Sample location |
|  | Sample type |
|  | Sample volume |
|  |  |
|  |  |
| **Analytical info** | Sample storage |
|  | Sample pretreatment |
|  | Person analysing |
|  | Sample divided (describe parts) |
|  | Sample analysis done |
|  | Sample photo done |
|  | Sample ID done |

## Sheet 2 – Classification template

The classification template consists of different categories that are organized with roll-down menus for each descriptive classification section. The categories can be adjusted in their corresponding work sheets.


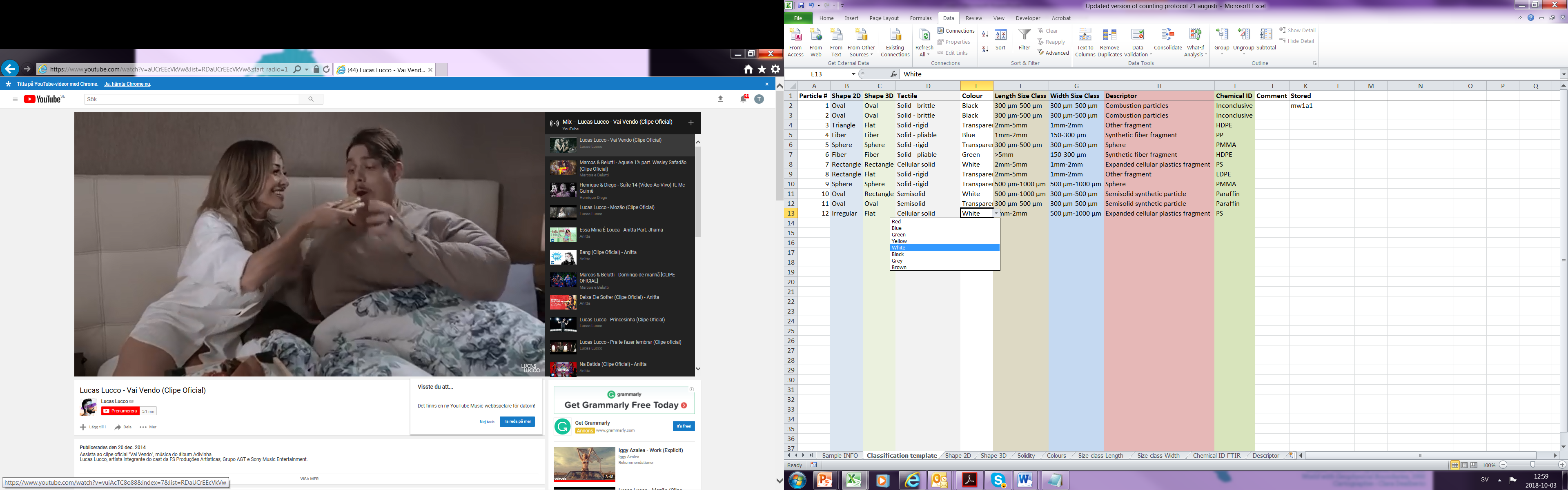


**Figure 2.** Outline of the classification template

### Shape parameters

Shape parameters are a useful method for categorizing particles. In addition for providing information about the particles, the shape could be useful from a toxicological perspective and to understand potential sources. The shapes can also be used, together with size and knowledge of the chemical composition, be used to calculate approximate volumes. Here the shapes have been split in 2D and 3D descriptors. The suggested shapes are: fiber, triangle, rectangle, square, oval, sphere, and irregular. As a high percentage of the particles are fragments the shapes will not be perfect triangles or squares, the shape descriptor will therefore often be an approximation, as illustrated in Figure 1 and in the examples provided in the end of the document. The 3D descriptors also include flat particles.

### Tactile parameters

One important benefit of particles in this size range is that you can touch them – make use of that. Mineral particles can for example be easier distinguished from plastic particles through scratching the surface. Semisolid synthetic particles are also easier to identify through applying pressure to the particle. Solidity in this context is not always straightforward, which we have covered more in depth in (Hartmann et al., 2019). Here it is simplified as solid, semisolid and non-solid particles. Particles defined as solid are then further divided into rigid (i.e. won’t break, bend or adapt to normal tweezer pressure), pliable (will bend easily without breaking, e.g. plastic bag), brittle (breaks easily), elastic (will respond to pressure but regain its shape as the pressure is released) and cellular (expanded plastics with cellular structures). Semisolids can for example be paraffin particles and non-solids could be oils.

### Colour parameters

Colours are useful to distinguish between as different colours may be more or less attractive to different organisms. They can also help reveal colour biases with the analyst. It should however be noted that colours might change and that several types of plastics would share similar colours, even so the colour can in some cases help distinguish between different specific sources. Here the colours red, blue, green, yellow, white, black, grey, brown and transparent are included. Other colours could of course be added but to avoid subjectivity a colour palette, as suggested in (Hartmann et al., 2019) could be used.

### Length and Width

Here length is measured as the longest length and width as the perpendicular width as illustrated in step two of Figure 1. Alternatively Ferret max and min could be used.

### Descriptor

The descriptor category is a short description of common types of particles. Other categories can be added but the analyst should strive for objective descriptors and avoid descriptors that hint to the origin of the particle. The descriptors used here include natural fiber fragments and synthetic fiber fragments. In samples above 300 µm these categories can rarely be quantified as most fibers will have a smaller width than the mesh, but occasionally thicker fibers are caught in the trawl.

Expanded cellular fragments are synthetic fragments that have air-filled cellular structures. Styrofoam is a commonly encountered type but similar fragments are also found that are polyethylene and polyurethane and other polymer types are also possible.

The descriptor “Other fragments” includes films and particles. Some analysts might chose to make further distinctions, for example between foils and other types of fragments, but these categories often overlap so here the categories that are used are simplified to keep the protocol clear and unambiguous. To use a more neutral differentiation between them we therefore suggest that they are sorted by the tactile section as either rigid or pliable.

Spheres include pellets, microbeads and other particles with a spherical shape. Semisolid synthetic particles include paraffin and polyethylene glycols with higher molecular weight. In the protocol we have also included combustion particles, oil, fat, metallic and others.

### Chemical ID

This option in the protocol is in case further analyses, for example with FTIR or Raman, are performed.

### Examples


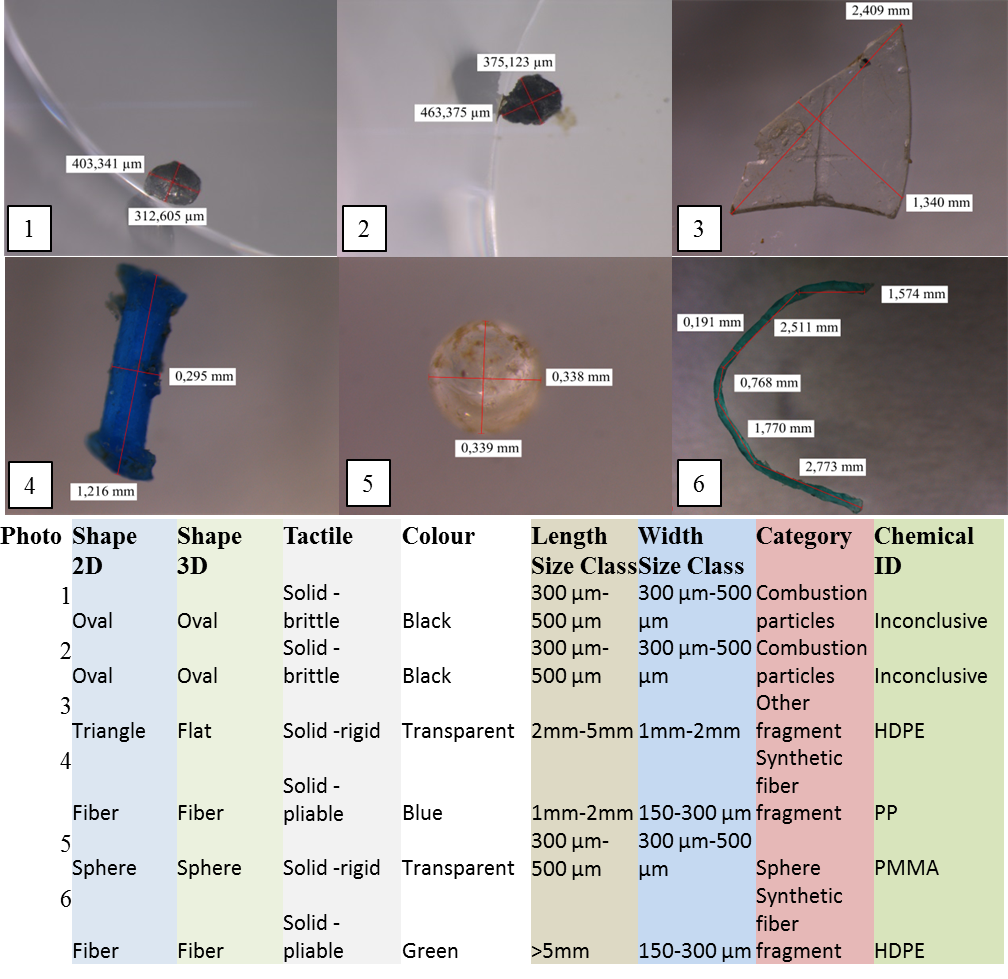


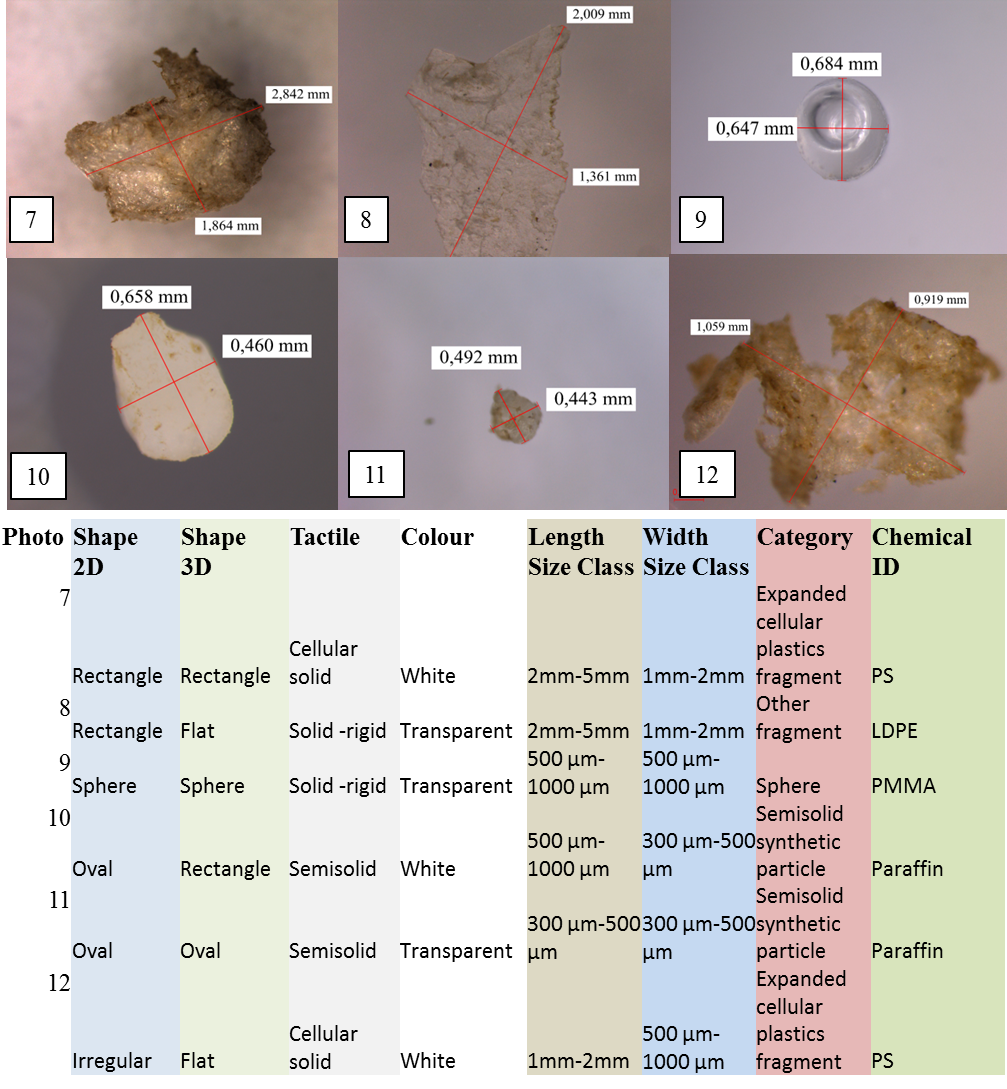


The collective community of microlitter research should also strive for a harmonization of identification parameters for comparable classification. The authors would welcome feedback to update the presented open source protocol, which is intended to be available for other users to expand upon and add classification parameters, or classification variables, appropriate for their samples / sizes.

# References

GESAMP (2019). Guidelines or the monitoring and assessment of plastic litter and microplastics in the ocean (Kershaw P.J., Turra A. and Galgani F. editors), (IMO/FAO/UNESCO-IOC/UNIDO/WMO/IAEA/UN/UNEP/UNDP/ISA Joint Group of Experts on the Scientific Aspects of Marine Environmental Protection). Rep. Stud. GESAMP No. 99, 130p.
